# Generation of Stable Induced Pluripotent Stem-like Cells from Adult Zebra Fish Fibroblasts

**DOI:** 10.7150/ijbs.34010

**Published:** 2019-08-24

**Authors:** Liangyue Peng, Yonghua Zhou, Wenting Xu, Minggui Jiang, Huajin Li, Mindi Long, Wenbin Liu, Jinhui Liu, Xiaoyang Zhao, Yamei Xiao

**Affiliations:** 1 State Key Laboratory of Developmental Biology of Freshwater Fish, Hunan Normal University, Changsha, 410081, China; 2 College of Life Sciences, Hunan Normal University, Changsha, Hunan, 410081, China; 3 School of Basic Medical Sciences, Southern Medical University, Guangzhou, 510515, China; 4 College of Biology and Environmental Engineering, Changsha University, Changsha, 410081, China

**Keywords:** induced pluripotent stem cell, doxycycline, zebra fish, fibroblast, lentiviral delivery system

## Abstract

Induced pluripotent stem (iPS) cells provide a powerful platform for the study of development, regeneration, and disease. Although many stable iPS cell lines have been established for mammals, few attempts have been made to induce iPS cells in nonmammalian species. Because of technical advantages over other vertebrates on stem cells, induced pluripotent stem cells from fish could be of value for research. In this paper, stable iPS-like cell lines were generated from adult zebra fish fibroblasts by combining the doxycycline inducible lentiviral delivery system and chemical treatment. RT-PCR analysis, alkaline phosphatase staining, and immunofluorescence indicated that adult zebra fish fibroblasts were successfully reprogrammed into iPS-like cells (ziPSCs). The ziPSCs exhibited stable growth and manifested many features of fish embryonic stem cells with pluripotency in vitro and in vivo. Because of easy maintenance, the developed technology in this study for generating zebra fish iPS-like cells could be extended to investigating other genera of fish.

## Introduction

Fish as models have several technical advantages compared with other vertebrates, such as higher fecundity; large, transparent embryos; and rapid development. Zebra fish is a small aquarium fish with high genetic conservation compared with the human genome and is regarded as an excellent vertebrate model system to study the genetics underlying development, normal body function, and disease 1. With regard to pluripotent stem cell research 2-7, zebra fish was one of the first nonmammals to have embryonic stem cells (ESC) successfully cultured in vitro 8. Owing to the inherent transparency, zebra fish embryos are proposed as a unique model for studying the dynamic aspects of stem cell development and differentiation 9,10.

Induced pluripotent stem (iPS) cells were first generated from mouse somatic cells by retrovirus-mediated induction with four core reprogramming factors: Oct3/4, Sox2, Klf4, and c-Myc 11. The establishment of iPS cells paved the way for basic and translational researches. In mammals, efficient and robust reprogramming techniques have been established and improved in the past 13 years, such as drug-inducible expression of defined factors 12,13, transfection of mRNA, miRNA, or protein as reprogramming factors 14-17, combination of chemical and genetic approaches 18-23, and direct reprogramming by small-molecule compounds 24,25. However, the iPS approaches mentioned above are largely limited to mammalian models, and few reports are available for nonmammalian species 26-28.

Rossello et al. provided the first evidence that mammalian transcription factors could be used to generate iPSCs from zebra fish embryonic fibroblasts 26. However, for the zebra fish system, no stable iPS-like cell line has ever been generated from the somatic cells of adult fish in feeder-free condition. In this study, we established iPS-like cell lines from zebra fish fibroblast cells by doxycycline (DOX) induced Yamanaka factors. Primary iPSC colonies were alkaline phosphatase (AP) positive. And the iPSCs maintained pluripotency after long-term culture in a modified culture condition. The technology developed in this paper provides a useful tool for research on other genera of fish.

## Materials and Methods

### Ethics statement

Those who conducted experiments with animals were licensed after attending a training course on laboratory animals provided by the Institute of Experimental Animals, Hunan Province, China. Procedures were conducted in compliance with the regulations of the Administration of Affairs Concerning Experimental Animals for the Science and Technology Bureau of China.

### Fish

All the experiments were conducted strictly in accordance with the Guidelines for the Care and Use of Laboratory Animals of the National Advisory Committee for Laboratory Animal Research in China and were approved by the Animal Care Committee of Hunan Normal University. Zebra fish (Danio rerio) were maintained at the State Key Laboratory of Developmental Biology of Freshwater Fish, College of Life Sciences, Hunan Normal University, as described previously 29.

### General cell culture

Zebra fish fin fibroblasts (zFFs) were derived from the caudal fin (1-year-old). For the primary caudal fin cell culture, fish were anesthetized with 100 mg/L MS-222 (Sigma-Aldrich, USA) before dissection. Caudal fin (~0.2 cm^2^) was minced with fine scissors and washed three times with sterile phosphate buffered saline (PBS) after a quick rinse with 70% ethanol. The tissue was placed in a new dish without medium for 15-30 min and then grown in the zebra fish fibroblast culture medium (ZF medium, see below for composition) for later culture in an incubator. For the primary midblastula stage embryonic fibroblast (zMEF) and primary somite stage embryonic fibroblast (zSEF) cell cultures, zebra fish embryos at the midblastula stage (4 h after fertilization) and the three-somite stage (10 h after fertilization) were first collected and then transferred to sterile PBS. After sterilization with 70% ethanol for 15 s and several rinses with PBS, the chorion and yolk were removed from the embryos by fine forceps. Consequently, the desired cell masses were released, and single cells were obtained by gentle pipetting. The cells were plated on gelatin-coated 96-well tissue culture plates with 100 μL of ZF medium.

All three types of zebra fish fibroblasts were cultured in ZF medium, which was composed of Dulbecco's modified Eagle's medium (DMEM) supplemented with 100 U/mL penicillin, 100 µg/mL streptomycin (Invitrogen, Carlsbad, CA, USA), 10% fetal bovine serum (FBS, Invitrogen), 2.5% fish serum (common carp), 0.1% 2-mercaptoethanol (2-ME, Invitrogen), 1 mM sodium pyruvate (Invitrogen), and 1 mM nonessential amino acids (Invitrogen). The cells were grown in 2.5% (v/v) CO_2_ at 28°C, passaged every 2 or 3 days. For the preparation of fish serum, whole blood was drawn from the tail vein of common carp (*Cyprinus carpio*) with a syringe. The extracted blood was placed on ice for approximately 6 h. The chromatograms were collected and centrifuged at 3500 x g for at least 15 min at 4°C. The supernatant was collected and filtered through a 0.22-μm filter on a clean bench and then stored at -20°C after being dispensed.

### Transduction of cells and ziPSCs culture

The lentiviral vectors TetO-FUW-OSKM and FUW-M2rtTA (Addgene) were used to reprogram the zFFs and zSEFs. After reaching 70% confluency, the 293T cells were digested by 0.05% trypsin for 3 min and then centrifuged at 1000 x g for 5 min. The collected cells were resuspended, counted with a hemocytometer, and seeded into a petri dish coated with 0.2% sterile gelatin. Virus transfection was conducted after culture for 24 h in a cell incubator at 37°C and 5% CO_2_ saturation. The cell culture medium was replaced with 5 mL of fresh prewarmed Opti-MEM^®^ Reduced-Serum Medium (Cat. No. 31985088; ThermoFisher, USA) a half hour before transfection. Virus transfection was performed according to the protocol of a Lipofectamine^TM^ LTX Reagent kit (Cat. No. 15338100; ThermoFisher). After transfection for 12 h, the culture medium was discarded, and 6 mL of culture media containing10% SP-free FBS was added to each petri dish. After 48 h, culture medium containing virus was collected and filtered through a 0.45-μm filter, for direct use for transduction or concentrated with an ultrafiltration tube (7,000 rpm for 30 min at 4°C; Cat. No. UFC910096; Millipore, Japan). (Note: fresh virus should be used to ensure efficient virus transduction).

Approximately 10^5^ fibroblasts were seeded on plastic in 35-mm culture dishes and infected with 20 µL of fresh virus (multiplicity of infection: 1.6-4 pfu/cell), in the presence of polybrene (8 µg/mL; Sigma-Aldrich, USA). Twenty-four hours after transduction (if necessary, fibroblasts were transduced twice to increase transduction efficiency), the cells were passaged onto matrigel and grown in the ZF medium plus 2 µg/mL doxycycline (Sigma-Aldrich, USA), 10 ng/mL bFGF, 0.5 μM MEK inhibitor (PD0325901; Stemgent), 0.5 μM ALK5 inhibitor (A-83-01; Stemgent), 3 μM GSK3β inhibitor (CHIR99021; Stemgent), and 1000 U/mL leukemia inhibitory factor. After transduction, the cells were subcultured when the cell morphology changed and colonies first observed. Then, the cells were passaged onto 0.2% gelatin-coated dishes and grown in ziPS medium (see below for composition). The ESC-like colonies with a typical dome shape were picked out and then expanded in the ziPS culture medium.

The ziPSCs were cultured on 0.2% gelatin-coated plates with ziPS medium, which was composed of DMEM supplemented with 7.5% FBS, 2.5% fish serum, 0.1 mL of zebra fish embryo extract (100 embryos/mL), 0.1% 2-ME, 1 mM sodium pyruvate, 1 mM nonessential amino acids, 100 U/mL penicillin, 100 µg/mL streptomycin, 10 ng/mL bFGF, 0.5 μM MEK inhibitor, 0.5 μM ALK5 inhibitor, 3 μM GSK3β inhibitor, and 1000 U/mL leukemia inhibitory factor. Cells were cultured with 2.5% (v/v) CO_2_ at 28°C. After reaching 90% confluency, the cells were replated at the split ratio of 1:2. For the preparation of zebra fish embryo extract, a total of 100 midblastula-stage embryos (12 hours post-fertilization) were collected and placed in an EP tube on ice. To each EP tube, 1 mL of cold PBS buffer was added, and the embryos were mashed with a glass rod on ice and centrifuged at 3500 x g at 4°C for at least 20 min. The supernatant was collected and filtered through a 0.22 μm filter. PBS was then added to a final volume of 1 mL/100 embryos, and fish embryo extracts were stored at -20°C.

### Production of zebra fish Oct4 and Nanog recombinant proteins and their antibodies

A cDNA corresponding to the 300 amino acids of Oct4 was inserted between the EcoRI and XhoI sites in a pET-32α (+) vector (Cat. No. GP0801; Genloci, China) and fused to a His tag. Similarly, a cDNA encoding the 315 amino acids of Nanog was also cloned into a pET-32α (+) vector. The recombinant fusion proteins were expressed in *E. coli* (BL21 (DE3) (Cat. No. M16054; Transgen, China)) and were purified using a Mag-Beads His-Tag Protein Purification Kit according to the manufacturer's protocol (Cat. No. C650033-0025; Sangon, China). To generate polyclonal antibodies against Oct4 and Nanog, 5 mg purified protein was emulsified in complete Freund's adjuvant (Cat. No. F5506-6X10ML; Sigma) and injected into the lymph of rabbits. After 15 days, the rabbits received a subcutaneous booster with the same amount of emulsified protein (three injections in total). Ten days later, antisera were collected and stored as aliquots at -20°C.

### Immunofluorescence

Cells were fixed in 4% paraformaldehyde for 30 min at 25°C and blocked with 2% BSA for 1 h. Primary antibodies of the following markers were used: Oct4 antibody (1:100), Nanog antibody (1:100), Sox2 antibody (1:100; Cat. No. GTX627404; GeneTex USA), and Nstin antibody (1:100; Cat. No. ab92391; Abcam, USA). The fluorescently labeled secondary antibodies anti-mouse IgG (for the anti-Sox2 antibody) and anti-rabbit IgG (for the antibodies of anti-Nstin, anti-Oct4, and anti-Nanog) were purchased from Jackson Lab (Sacramento, CA, USA). Nuclei were stained with Hoechst33342. Fluorescence was imaged using a Zeiss LSM510 confocal microscope (Carl Zeiss AG, Oberkochen, Germany).

### Reverse transcription-polymerase chain reaction

Total RNA was isolated using Trizol reagent (Invitrogen). Five micrograms of total RNA was treated with DNase I to remove potential genomic DNA contamination by using a DNA Free RNA kit (Zymo Research, Orange, CA, USA). One microgram of DNaseI-treated RNA was reverse-transcribed using a First-Strand Synthesis kit (Invitrogen) and ultimately resuspended in 100 μL water. Then, the first-strand cDNAs were used as templates for RT-PCR. The PCR primers are listed in Table 1.

### Karyotype analysis

The ziPS-like cells were treated with 0.2 µg/mL colchicine for 4-6 h and then with a hypotonic solution of 0.075 M KCl for 30 min. The cells were fixed twice with cold Carnoy's fixative (methanol:glacial acetic acid, 3:1, v/v) for 15 min each time, and drops of cell suspension were spread on cold slides, which were air-dried for 2 h. The chromosomes were stained with 5% Giemsa solution for 20 min. More than 100 metaphases were examined for each cell line.

### Embryoid body formation

Embryoid body (EB) was generated using the method of the hanging drop 30. The ziPS cells were inoculated at a density of 10^5^ cells/mL in a 12-well plate using ziPS medium without LIF, cytokines, chemical inhibitors, and mercaptoethanol. The EBs were collected and placed again into a gelatin-coated well. The ziPS medium was refreshed every 2-3 days.

### Fluorescent dye labeling and chimera formation

The ziPSCs were first trypsinized and collected at density of 10^7^ cells/mL in Diluent C (Sigma). The cells were stained with the fluorescent dye PHK26 (red; Sigma) for 5 min. Serum was added to terminate the labeling reaction, and the unbound dye was removed by repeatedly washing with PBS.

Chimera formation was evaluated through the transplantation of PHK26-labeled ziPS cells into the midblastula stage of zebra fish embryos, as previously described 31. The labeled ziPS cells were suspended in PBS. Approximately 200-300 donor cells (equal ratio of cells) were injected into the deep cell layer of each embryo. The recipient embryos were incubated in E3 medium at 28.5°C after injection and were monitored under a fluorescent microscope.

### Cell transplantation in adult zebra fish

The ziPSCs were labeled with PKH-26. The recipient pearl danio (*Danio albolineatus*), anesthetized with 0.02% Tricaine, were placed on a culture dish, and the ziPSCs were injected directly into the abdominal cavity with 10 μL of cell solution (approximately 1000 cells). The injected fish were placed in water for 5 h containing 10 ppm Tricaine. Ultimately, the fish were bred normally.

## Results

### Selection of fibroblasts for iPSC generation

The zebra fish fibroblast cell cultures were initiated using the conditions described by Hong et al. 9. The dissociated cells from the embryos of the zebra fish midblastula and segmentation stages and from the zebra fish caudal fin were seeded onto gelatin-coated multiwell plates with ZF medium (see also the section in Materials and Methods). The cells began to adhere to the well surfaces within 24 h and then formed clusters approximately 2 days post-culture. When culture reached 80% of the culture dish (4-6 days), cells were digested by 0.25% trypsin for 3 min and then replated at the splitting ratio of 1:2. The phenotype and the stable growth of fibroblast cultures were consistent during serial subculture (Fig. S1A).

To determine the pluripotency of short-term cultured zebra fish fibroblast cells, the gene expression patterns of some pluripotency factors and the activity of alkaline phosphatase (AP) were analyzed. The zMEF cells possessed AP activity (Fig. S1A), and some ESC marker genes were expressed (e.g., *oct4, sox2, lin28, and nanog*) (Fig. S1B). However, zSEF cells and zFF cells were negative for AP staining and none of the pluripotent genes were expressed.

### Generation of zebra fish iPS-like cells from embryonic and fin fibroblasts

**Figure 1:**
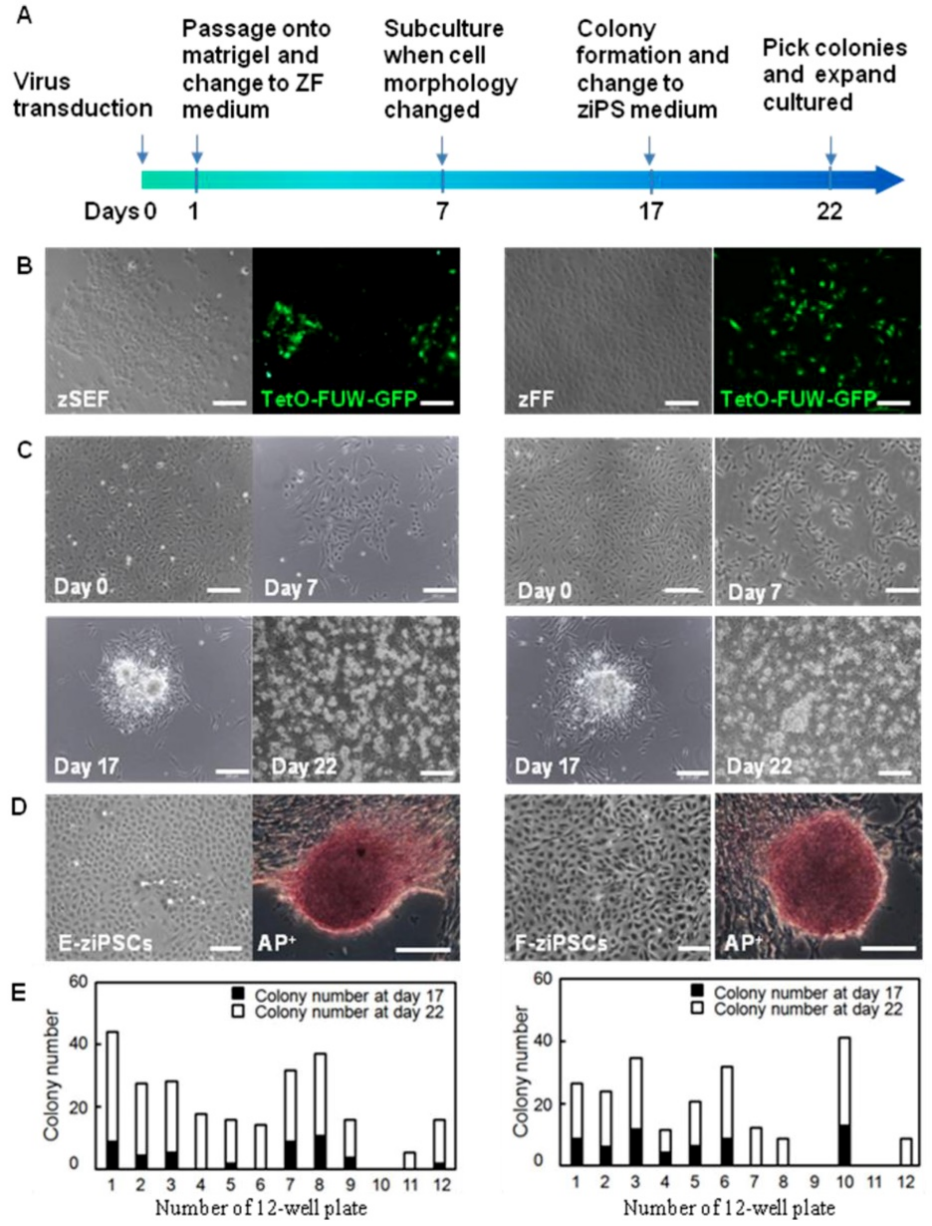
** Generation of zebra fish iPS-like cells from embryonic and fin fibroblasts. (A)** Workflow to generate zebra fish iPS cells (ziPSCs). **(B)** zSEF (left two panels) and zFF (right two panels) cells grown in ZF medium after transduction with TetO-FUW-GFP. **(C)** The time course and cell morphology of reprogramming in primary transduced zSEF (left four panels) and zFF (right four panels) cells with an optimized reprogramming system. **(D)** Zebra fish iPS-like cell line (right) and iPS-like colony with AP positive staining (left) derived from zSEF (E-ziPSCs, left two panels) and zFF (F-ziPSCs, right two panels) cells. **(E)** The number of colonies at day 17 (black columns) or day 22 (white columns) after transduction. The results were obtained from at least three independent experiments. Scale bars represent 200 μm.

As shown in Figure 1A, a reprogramming protocol was developed (see also the section in Materials and Methods for details). The lentiviral vectors of TetO-FUW-GFP and FUW-M2rtTA were used to transduce the zSEFs and zFFs. After transduction, the zFFs and zSEFs were grown in the ZF medium. The GFP was expressed in most cells, indicating efficient viral transduction and transgene induction (Fig. 1B).

As shown in Figure. 1C, the zSEFs and the zFFs with good growth status were selected for viral transduction. 7 days after transduction, the cell morphology changed a lot and the colonies could be firstly observed. Approximately 17 days after transduction, the ESC-like colonies were domed with a clear boundary (Fig. 1C), similar to the result on the zebra fish ESCs in 9. The ziPSCs stained positive for the pluripotent surface marker ALP (Fig. 1D). The ESC-like colonies were picked out at 17 and 22 days, and transferred to 12-well plates for further expansion. The zebra fish ES-like cells (named zebra fish iPS cells or ziPSCs) could be expanded in the ziPSCs medium in a feeder-free condition (Fig. 1D). To further understand the efficiency of colony formation, the number of colonies was calculated at 17 and 22 days after transduction (Fig. 1E). For each 12-well plate on day 22, the average colony number of the ziPSCs derived from zSEF and zFF was 265 and 221, respectively.

### Characterization of zebra fish iPS-like cells

**Figure 2:**
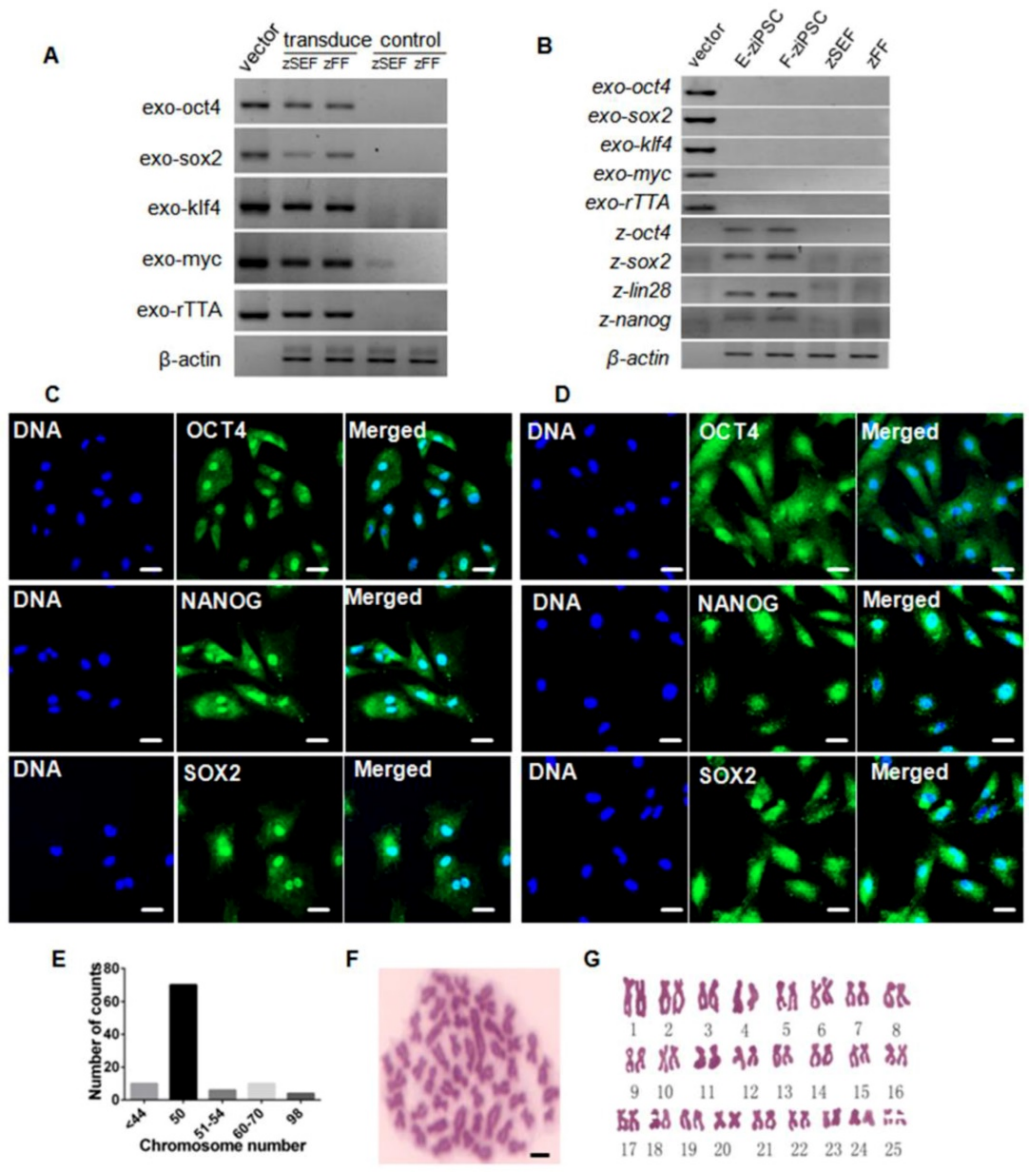
** Characterization of zebra fish iPS-like cells. (A)** RT-PCR analysis of exogenous *rTTA, oct4, sox2, klf4* and *c-myc* in zSEFs and zFFs at 7-day post-transduction. At least three independent experiments were conducted for these results). **(B)** RT-PCR analysis of endogenous* oct4, sox2, lin28*, and *nanog* in the E-ziPSCs (passage 20) and F-ziPSCs (passage 18) (At least three independent experiments were conducted for these results). **(C)** Immunofluorescence staining of Oct4, Nanog, and Sox2 in E-ziPSCs (passage 20) (Scale bars represent 20 μm). **(D)** Immunofluorescence staining of Oct4, Nanog, and Sox2 in F-ziPSCs (passage 18) (Scale bars represent 20 μm). **(E)** Distribution of chromosome numbers among 100 F-ziPSCs metaphases (passage 18-30). **(F)** A metaphase plate of the chromosomes of a diploid F-ziPSC (2n = 50) after Giemsa staining (Scale bar represents 10 μm). **(G)** Diploid karyotype of an F-ziPSC (homologous chromosomes were paired according to their sizes).

The zebra fish iPS-like cells were characterized by immunofluorescence staining with pluripotency markers, including Oct4, Sox2, and Nanog. At 7-day post-transduction, the expression of the three marker genes was robustly induced (Fig. S2). And exogenous reprogramming genes in the zSEFs and zFFs were analyzed by RT-PCR, which showed actively transcription (Fig. 2A). We picked up 10 colonies from each of zSEFs and zFFs at 22 day after transduction, and respectively established three and two stable ziPSC lines such that more than 30 passages were expanded. Two ziPSC lines, named E-ziPSCs (derived from the zSEF) and F-ziPSCs (derived from the zFF), were selected for further RT-PCR analysis. The exogenous *r*TTA and the four Yamanaka factors were silenced in the two ziPSC lines, whereas the endogenous *oct4, sox2, lin28,* and *nanog* were activated (Fig. 2B). These results suggested that the reprogramming was completed at the molecular level in the ziPSCs possessing the characteristics of pluripotent stem cells. The E-ziPSCs and F-ziPSCs were further characterized by immunofluorescence staining with Oct4, Sox2, and Nanog. As shown in Figure 2, the expression of the three marker genes was active in these ziPSCs (Fig. 2C, D). To determine the chromosome composition of the ziPSCs, the cytogenetics of the F-ziPSCs were analyzed by examining more than 100 metaphases. Among them, 71% of the cells were diploid (44-54 chromosomes), 16% were hypodiploid (<44, 51-54 chromosomes), 10% were triploid (60-70 chromosomes), and 3% were tetraploid (∼98 chromosomes) (Fig. 2E-G).

### Differentiation potential of zebra fish iPS-like cells *in vitro*

**Figure 3:**
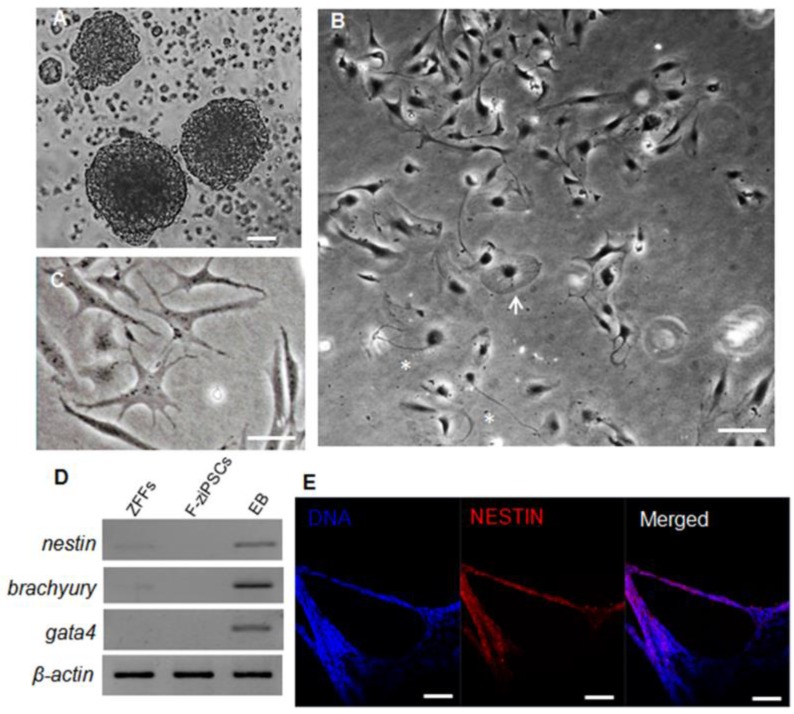
** Differentiation potential of zebra fish iPS-like cells in vitro. (A)** Embryoid bodies (EBs) derived from F-ziPSCs (passage 15). **(B)** Differentiated cell types from the EBs after attaching to a culture plate, showing a flat cell (arrow) and a neuron-like cell (asterisk). **(C)** Star-shaped cells. **(D)** Results by RT-PCR analyses of the three differentiation markers (*nestin*, *brachyury*, and *gata4*) for the three germ layers (At least three independent experiments were conducted for these results). **(E)** Nestin expression in the EBs formed from F-ziPSCs (passage 15) determined by immunofluorescence. The scale bars in (A-C) represent 50 μm and that in (E) represents 200 μm).

To determine the differentiation capacity of the ziPSCs, *in vitro* EB was generated using hanging drop method (Fig. 3A). When seeded at low cell densities in the ZF medium, the F-ziPSCs differentiated into various types of specialized cells (Fig.3S), which including flattened cells (Fig. 3B and Fig. S3B), star-shaped cells (Fig. 3C and Fig. S3C), and neuron-like cells (Fig. 3B and Fig. S3D). The marker genes of three germ layers were detected, and *nestin* (ectoderm), *brachyury* (mesoderm), and *gata4* (primitive endoderm) showed high expression in EBs derived from F-ziPSCs but no any exists in F-ziPSCs (Fig. 3D). The EBs were also positive for the ectoderm marker Nestin by immunofluorescence staining (Fig. 3E). These results indicated that the ziPSCs possessed the multi-linage differentiate capacity *in vitro*.

### Pluripotency of zebra fish iPS-like cells *in vivo*

**Figure 4:**
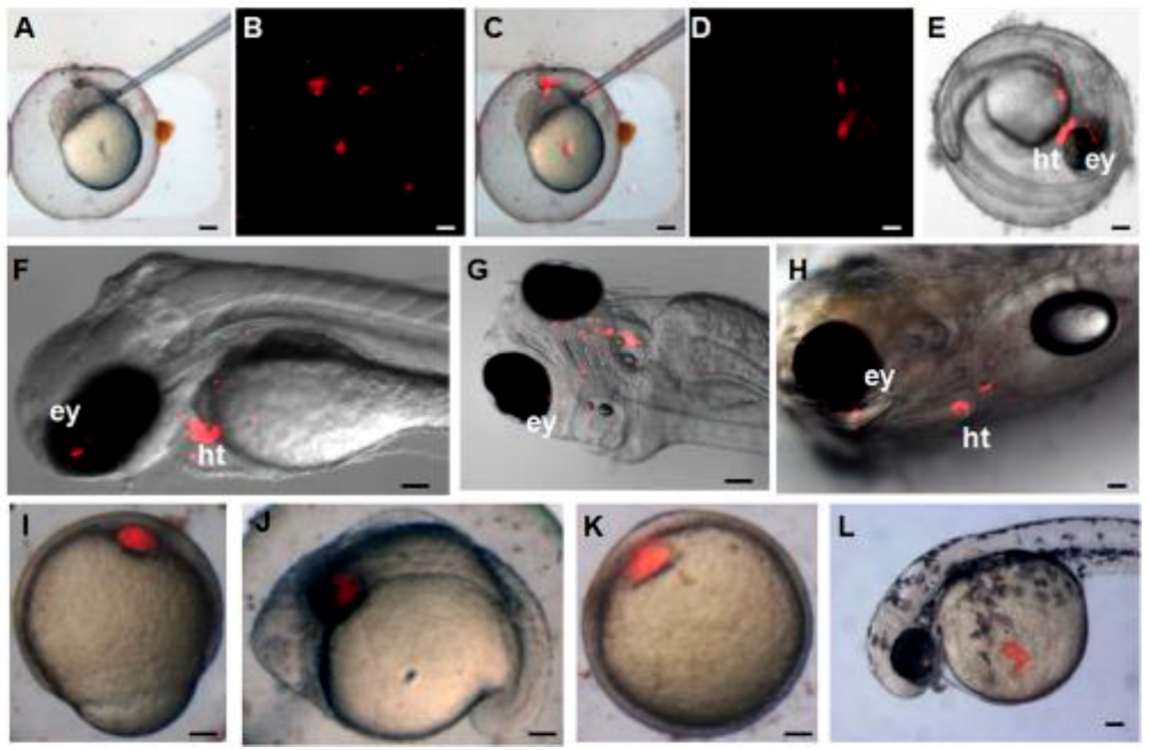
** Differentiation potential of zebra fish iPS-like cells *in vivo*.** PKH26-labeled F-ziPSCs (passage 22) and PKH26-labeled zFF cells (passage 10) were transplanted with approximately 300 cells to host blastulae. The resultant chimeras were analyzed by microscopy. For the PKH26-labeled F-ziPSCs (red): **(A)** bright field micrograph, **(B)** red fluorescent micrograph, **(C)** merge between bright and fluorescent optical fields, and **(D-H)** different distributions of PKH26-labeled donor cells in different stages, in which the chimeras were analyzed by microscopy at 2 **(D-G)** and 5 days post-fertilization (H) (ey, eye; ht, heart). For the PKH26-labeled zFF cells (red): **(I-L)** the zFF cells did not migrate. The scale bars in A-E and I-L represent 200 μm and those in F-H represent 300 μm.

The pluripotency of ziPS-like cells *in vivo* was tested by chimera formation. The ziPS-like cells were labeled with a fluorescent dye (PHK26) and then transplanted into the zebra fish blastula hosts (Fig. 4A-C). As shown in Table 2, approximately 83% of the host blastula embryos survived in the three experiments. Our data showed that 58% embryos and 29% hatched fries were positive for PKH26. The ziPS-like cells exhibited an overlapping distribution in chimeric embryos (Fig. 4D, E). Particularly, in 2-5-day-old chimeric embryos, the ziPS-like cell derivatives were found in a wide variety of tissues, such as head, eye, trunk, tail, and yolk sac (Fig. 4D-H). In the control groups, we saw that some PKH26-labeled cells survived after around 300 PKH26-labeled zFF cells were injected into each host blastulae. However, the injected fibroblasts could not migrate during the development of the recipient embryos, and the volume of the injected fibroblast cluster did not become larger (Fig. 4I-L).

**Figure 5:**
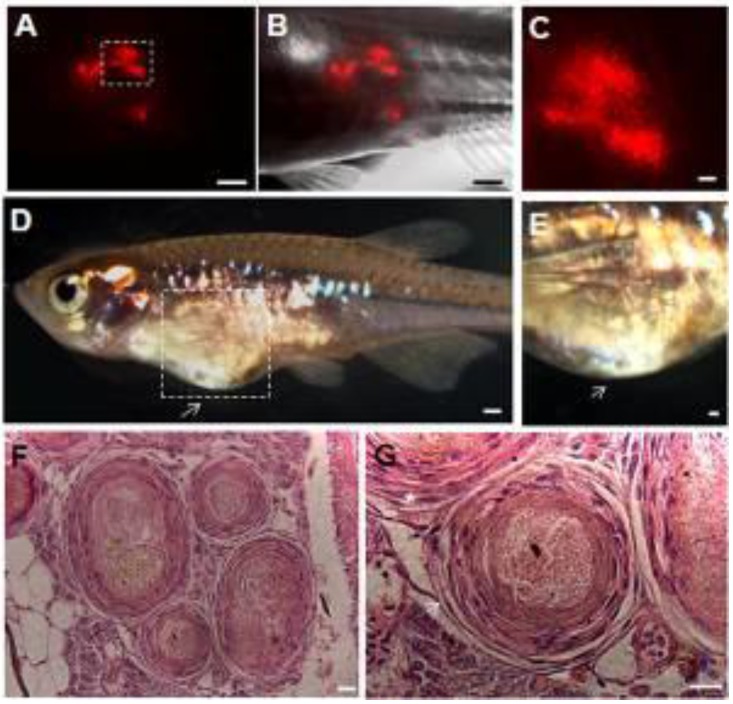
** Teratoma formation *in vivo*.** PKH26-labeled F-ziPSCs (passage 22, red) were transplanted with approximately 1000 cells to host adult male pearl danios (*D. albolineatus*), and the resultant teratomas were analyzed by microscopy at four weeks after injection **(A-C)**. After 10 weeks post-injection, the teratomas were photographed **(D, E)**, and histological analysis was conducted with hematoxylin and eosin staining **(F, G)**. Of the 10 injected fish, teratomas were observed in two fish. (A) Red fluorescent micrograph. (B) Merge between bright and fluorescent optical fields. (C) A close-up of the boxed area in (A) to show the possible proliferation of PKH26-labeled cells. (D) Photograph of adult male pearl danio at 10 weeks after injection to show the teratomas (arrow). (E) Magnification of the boxed area in (D). (F) Various tissues in the ziPSC teratomas. (G) Histological section of teratoma including muscle for mesoderm (asterisk) and epithelium for ectoderm (arrow). The scale bars in (A-E) represent 500 μm and those in (F, G) represent 20 μm.

The capacity of ziPSCs to form teratomas *in vivo* was also examined. PKH26-labeled ziPSCs (10^3^) were subcutaneously injected into adult male *D. albolineatus.* The PKH26-labeled cells were first observed in the recipient fish under standard fluorescence microscopy 3 days post-injection. The number of the labeled cells increased significantly at four weeks post-injection (Fig. 5A-C). Teratomas formed in two out of the ten recipient fish at 10 weeks post-injection (Fig. 5D, E). Histological examination showed that the teratomas were associated with derivatives of the three germ layers (Fig. 5F, G). These results indicated that the ziPS-like cells were pluripotent* in vivo*.

## Discussion

In the past two decades, the zebra fish has been developed into a versatile platform for stem cell research because of the virtues of conserved stem cell regulatory pathways, superior imaging characteristics, and extraordinary regenerative capability, compared with mammals. Numerous types of stem cells have been studied in embryo and adult zebra fish 9, 10, 32-35. In this research, an optimized reprogramming system was established to derive zebra fish induced pluripotent stem cell-like (ziPS-like) cell lines from the embryonic and fin tissue. The ziPS-like cells displayed typical features of fish ESCs, described previously in several fish species 33, 36-39. The cells could self-renew after long-term culture and differentiate into various cell types both *in vitro* and *in vivo*. Thus, this research provides an efficient way to generate and maintain ziPS-like cells.

The reprogramming of somatic cells into pluripotent stem cells by Yamanaka factors is a slow process. The basic procedure has been variously modified to improve the efficiency of the iPSC production 14-19. The delivery of transgenic factors by lentiviral-mediated expression vectors can help to improve the reprogramming efficiency. A DOX-induced lentiviral delivery system can increase the production of iPSCs 12, 13, 40, 41.

Besides, chemical treatment can also be a strategy to improve the efficiency of reprogramming 18. For example, histone deacetylase inhibitors (valproic acid, trichostatin A, and suberoylanilide hydroxamic acid), DNA methyltransferase inhibitors, MAPK/ERK kinase inhibitor (PD0325901), ALK5 inhibitor (A-83-01), LSD1 inhibitors, glycogen synthase kinase 3 beta inhibitor (CHIR99021), and vitamin C can substantially increase reprogramming efficiencies 18, 42-45. Shimada et al. successfully generated canine iPSCs by lentiviral transduction and chemical inhibitors 22.

In this study, the combination of the DOX-inducible lentiviral delivery system and chemical treatment optimized the protocol of the induction system in zebra fish. This protocol successfully induced zebra fish fibroblasts to pluripotency, suggesting that the strategic combination used in this paper may be a good option for modifying the reprogramming system. Recent reprogramming studies show that small molecules can directly induce somatic cells to pluripotency in mammalian systems 24, 25, 46. Carp fibroblasts have also been successfully induced to pluripotency using similar protocols (unpublished data).

When the number of iPSC passages increases, the ability to differentiate may decrease 47. Rossello et al. showed in vivo incorporation and differentiation of cells in birds and fish, although no stable cell lines were obtained 28. As one of the primary contributions of this research, the ziPSC medium was optimized such that pluripotency was maintained after long-term culture. Specifically, the medium was a DMEM supplemented with MEK inhibitor, ALK5 inhibitor, GSK3β inhibitor, bFGF, LIF, FBS, carp serum, and zebra fish embryo extract. Notably, the important difference between the ziPSC culture medium in this study and the previously unsuccessful ones was the supplementation with zebra fish embryo extract and carp serum. The inclusion of embryo extract is also reported in the medium for culturing the ESCs of several fish 9, 33, 36, 37, 39, 48.

Early zebra fish embryo cells maintain pluripotency after multiple passages in culture 49. Xiao et al. concluded that pluripotency was maintained in a very narrow window in zebra fish embryos from zygotic genome activation to a brief moment after the oblong stage 35. In this study, fibroblasts from blastocyst stage embryos with the ZF medium retained pluripotency, but those cells from the somatic embryos or adults had no pluripotency after multiple passages in culture. Since caudal fin fibroblasts are more readily available than embryonic fibroblasts, particularly for endangered fish species, the fin fibroblasts are recommended for reprogramming of other fish genera.

## Supplementary Material

Supplementary figures.

## Figures and Tables

**Table 1:** Primers used in PCR application.

Genes	Forward primers	Reverse primers
*z-oct4*	ATTTCAACGGAATCACCCCC	TGAGCTGAGGGAATGTTTTGC
*z-sox2*	ACCCCGGAGGAAAACCAA	AGCCCAGTGTCATTCCCGGC
*z-lin28*	CGTCCATCAGAGCAAGCTTCA	CGATTTGCCCTGAGATCCTG
*z-nanog*	CCAAAAGGCCAAAGATGCAG	CCTGTTGCTCCAGCAAGCGTTT
*z-actin*	GGTATTGTGACCAACTGGGATG	TCCCATCTCCTGCTCAAAGTC
*ecto-oct4*	TTTTGACCTCCATAGAAGACACC	TTCTCCAACTTCACGGCATT
*ecto-sox2*	CAAGCAGGTGATGTTGAAGAA	AAGCGTGTACTTATCCTTCTTCAT
*ecto-klf4*	GGCACGGCCATTAACGGCACA	TGGTGGGTTAGCGAGTTGGA
*ecto-myc*	CTGGATTTCCTTTGGGCGTTGG	GTAGCGACCGCAACATAGGA
*ecto-rTTA*	GTAAATTGTCCGCTAAATTCTGG	AAGTGGGTATGATGCCTGTCC
*nestin*	ACTGTGTGGAGCTGGAGGTAG	TCCTGCCAGGCTTGAGCTGC
*brachyury*	CTGGCCCAAACTCAAACACCA	GGTGCCACATGGGTCCAGGC
*gata4*	CGTCTGGACACTCTCGATGGG	ACACTCCCGCCCTCGCAGAT

**Table 2:** Embryos injected with PKH26-labeled zebra fish iPS-like cells and fibroblasts.

Experi-ment	Donor cells	Passage number	Embryos Injected n	Embryos Survived n (%)	PKH26-positive Embryos n (%)	Hatched Chimeras n (%)
1	ziPSCs	10	254	214(84)	162(64)	72(28)
	fibroblasts	10	200	171(86)	143(72)	0
2	ziPSCs	22	123	102(83)	72(59)	40(33)
	fibroblasts	10	81	65(80)	31(38)	0
3	ziPSCs	22, 28	105	86(82)	45(43)	27(26)
	fibroblasts	10	96	80(83)	32(33)	0
Total	ziPSCs		482	402(83)	279(58)	139(29)
	fibroblasts		377	316(84)	206(55)	0
