# Supplementary material for: Generation of Stable Induced Pluripotent Stem-like Cells from Adult Zebra Fish Fibroblasts
Source: Int J Biol Sci. 2019 Aug 24;15(11):2340–9. doi: 10.7150/ijbs.34010 (PMC6775306; doi:10.7150/ijbs.34010)
Supplement: Supplementary file 1 — Supplementary figures. [file ijbsv15p2340s1.pdf]

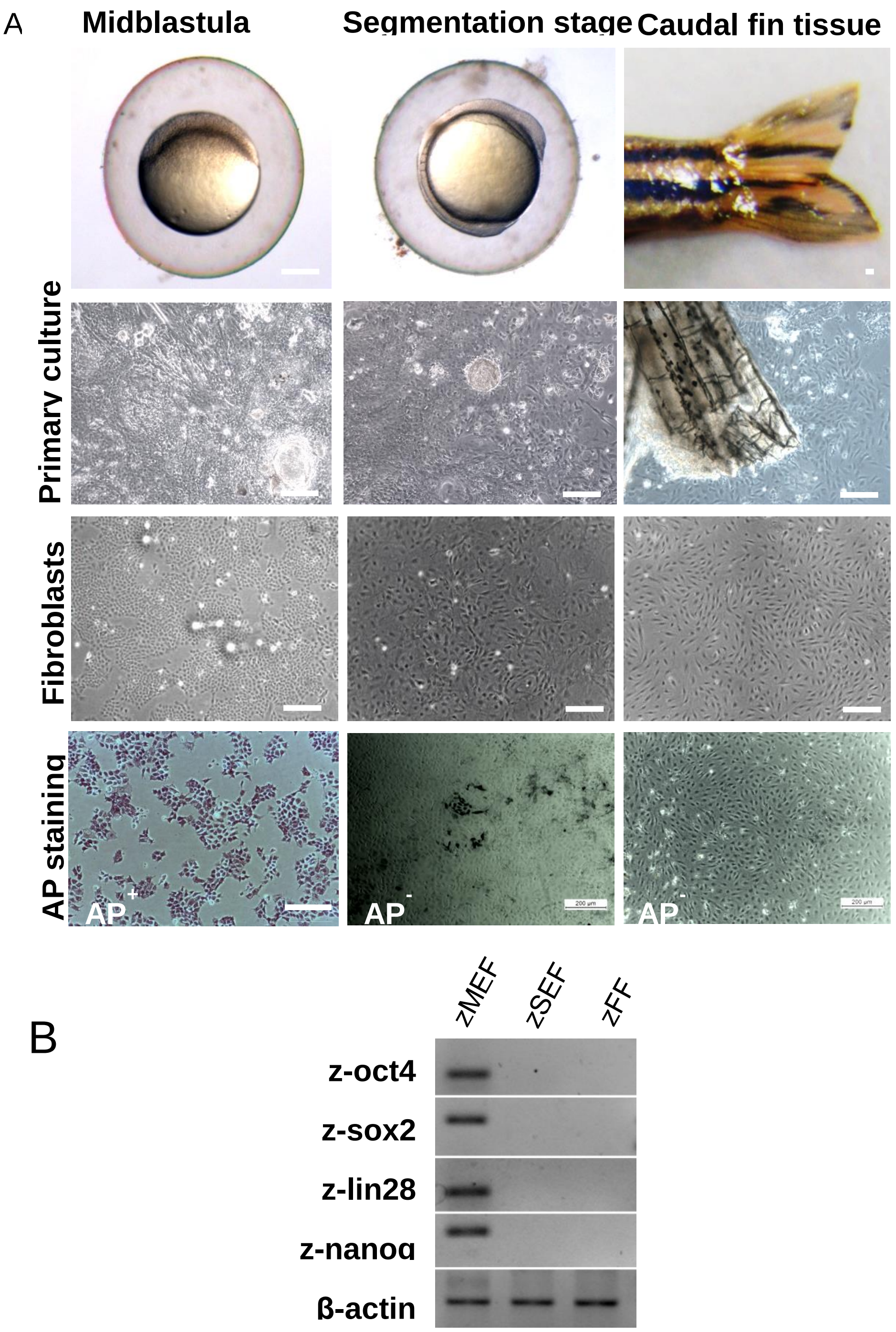

Figure S1. Characterization of 3 types of cultured zebra fish fibroblasts

(A) Culture of zebrafish fibroblasts from the midblastula embryos (zMEF), segmentation embryos（zSEF), and caudal fin tissue (zFF). zMEF at passage 5 showed AP staining positive. (B) RT-PCR analysis of ES cell-marker genes expression in the zMEF, not in the zSEF and zFF. Scale bars represent 200μm.

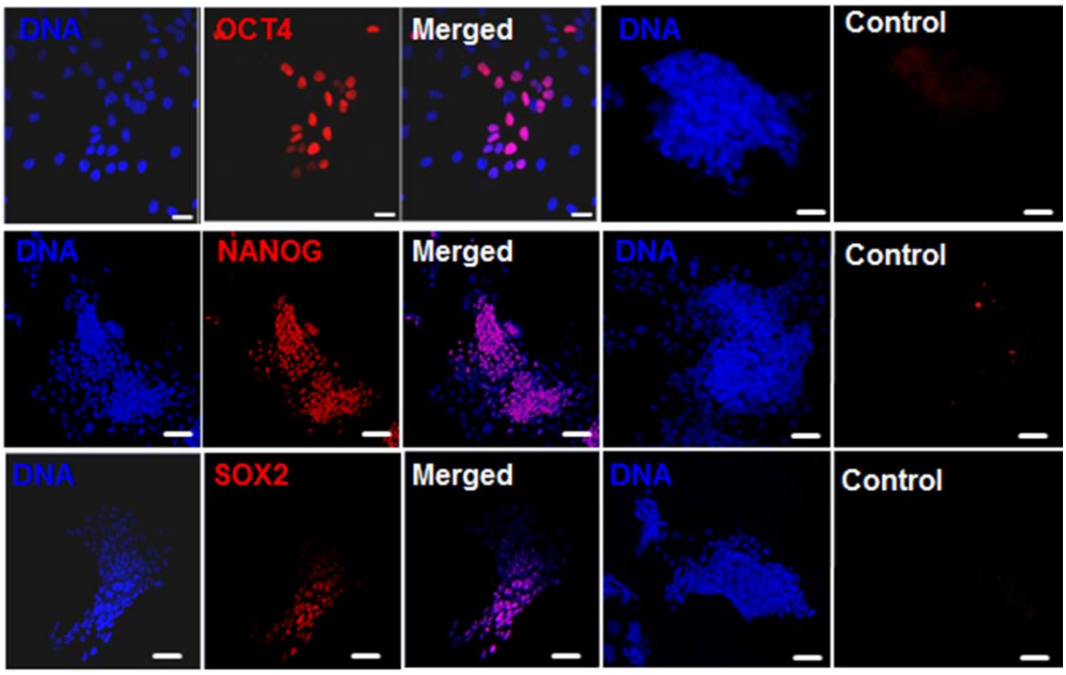

Figure S2. Immunofluorescence staining of 7- day post-transduction cells (derived from the zFFs) for pluripotency markers Oct4, Nanog, and Sox2. Scale bars represent 50 μm).

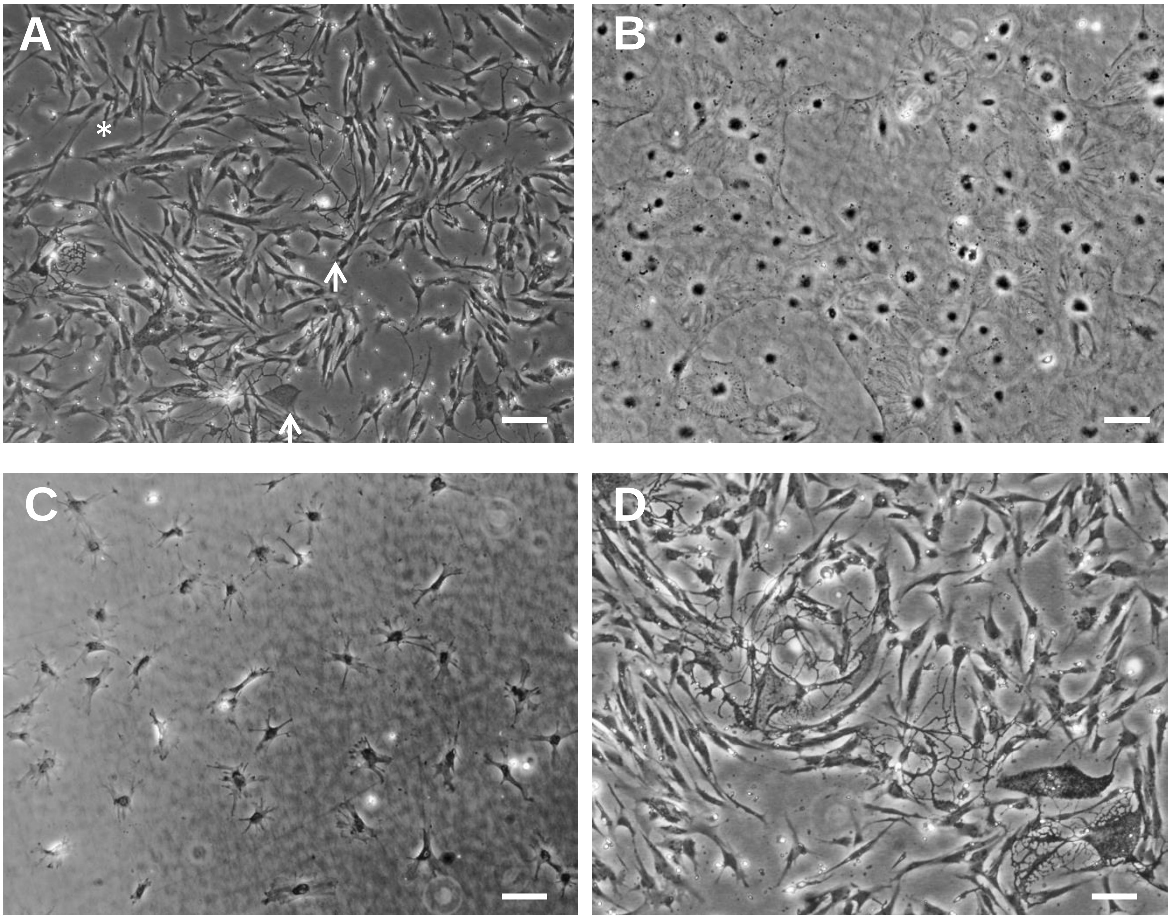

Figure S3. Differentiated cell types from embryoid bodies. (A) Differentiated (arrow) and undifferentiated (asterisk) cells. (B) Flat cells. (C) Star-shaped cells. (D) Neuron-like cells. Scale bar represents 200 μm.
